# Construction and application of product optimisation design model driven by user requirements

**DOI:** 10.1038/s41598-024-67406-x

**Published:** 2024-07-16

**Authors:** Zhigang Hu, Dongyi Jia, Xianling Qiao, Nan Zhang

**Affiliations:** 1 College of Design and Art, Shaanxi University of Science and Technology, Xi’an, 710021 China; 2 School of Art and Design, Shaanxi Fashion Engineering University, Xianyang, 712046 China

**Keywords:** Product design, User requirements, Programmatic decision making, Game modeling, Engineering, Applied mathematics

## Abstract

User requirements serve as the primary reference content in product design. The effective capture of crucial user requirements, followed by the development of a product technical solution aligned with these requirements, stands as a pivotal approach to enhancing design efficiency. In order to explore the problem of generating and decision-making of product technical solutions in the case of complex user demands, this study constructs a user requirements driven product optimization design model, which is used to complete the generation and decision-making of product design solutions in a more reasonable way. The model unfolds across three key stages: Firstly, a user requirements importance ranking system is crafted leveraging Kano Model and Pairwise Analysis. Next, employing the Functional Analysis System Techniques (FAST) theory and the Quality Function Deployment (QFD) theory, user requirements undergo transformation into technical solutions. Finally, these technical solutions are amalgamated into diverse technical combinations, with decisions facilitated by a game theory model to yield the optimal overall design solution. The new optimal design model reduces the influence of subjectivity and ambiguity in the process of user requirements analysis, increases the reliability of the transformation of user requirements into technical solutions, and improves the efficiency of the generation and decision-making of product design solutions under multiobjective situations. The model proposed in this study is exemplified through the development of a medicated bath water heater. Results indicate that the technical solution derived from the model surpasses similar products in terms of user satisfaction, thereby validating its feasibility.

## Introduction

With the diversity and complexity of user needs, their effective analysis and the proposal of corresponding product technology solutions are important issues faced by enterprises in product optimization and design^1^. In recent years, the methodological research on the transformation of user requirements into technical solutions in the conceptual design stage has centered on the analysis of user requirements and the transformation and selection of technical solutions^2^.

Research on requirement analysis has dealt with two main aspects: requirement establishment and requirement importance analysis. Requirement establishment mainly refers to organizing the user’s expectations of the product into a specific expression of requirements. Zhang et al.^3^ used SAS software to analyze web review data and patent knowledge in order to obtain user requirements, whereas Qiao et al.^4^ categorized products into three levels (performance, interaction, and appearance), introducing the double-diamond model to mine and analyze user requirements and establishing user requirements more comprehensively from the perspective of product functions. In requirement importance analysis, the requirements are evaluated on the basis of user satisfaction and then ranked in order of importance. Tang et al.^5^ used fuzzy clustering combined with the Kano model to construct a personalized demand hierarchy model in order to obtain demand weights. Chen et al.^6^ combined KJ with FAHP to quantify and rank the weights of user requirements for nucleic acid detection robots. The above methods could effectively establish user requirements to determine the design focus of a product, but they featured strong subjectivity in the screening of demand variables, importance assessment, etc., and a lack of quantitative support for the subsequent refinement and evaluation of technical solutions.

Studying the translation of needs into technical solutions, Li et al.^7^ proposed a design method based on the Kano and QFD (Quality Function Deployment) approaches to analyze the importance of technical indicators in the design of intelligent public facilities according to the preference of user requirements and provide design guidance based on high-importance technical indicators. Su et al.^8^, taking the division of user attributes as the premise, constructed a comprehensive 3D printed spinal orthosis design model combined with the AHP entropy weight method to determine the design elements and clarified the innovative direction of the technical program, in this case, the technical program is the sum of the technologies used in the design of the product. Although the above research provided an effective method for transforming user needs into technical solutions, the proposed technical solutions were relatively homogeneous, and there was little room for solution preference. Ye et al.^9^ investigated an innovative design method for camping chairs by combining Kano-QFD and FSB modeling and, based on quantitative analysis and user requirement analysis, carried out requirement function mapping, proposed multiple groups of design options, and made program decisions. Wang et al.^10^ presented a design solution optimization model based on the entropy weight approach and VIKOR method, which proposes a design solution set by transforming user requirements into design indexes, and incorporated the quantitative analysis of user requirements for design solution optimization. The above research extended the decision space of technical solutions, but problems such as a disconnection between user needs and technical solutions, a lack of logical rigor, and poor method generalization still exist.

In summary, although the above studies improved the effectiveness of translating user needs into technical solutions, however, several issues persist, as outlined below:

1. The influence of users’ subjective differences was ignored when determining demand importance, which led to poor objectivity in the demand importance assignment.
2. Uncertainty in delineating the importance of design elements led to bias in the allocation of resources to subsequent technical solutions, conflicting with user preferences.
3. The multiobjective attributes of the product were ignored in the technical solution selection stage, preventing the rational screening of the technical solutions from an objective perspective.

In order to address the above issues, this study proposes a product optimization design model integrating the Kano model, QFD, and game theory (KQGT). The main innovations and contributions of this study are as follows:

1. This study established a user requirements importance ranking system to mitigate subjective influences during the analysis of user requirements.
2. Establishes an effective process for the transformation of user requirements into technical solutions. The reliability of the transformation of user requirements into technical elements is improved, and technical solutions are proposed on this basis based on the technical elements. Among these components, the technical element represents a specific engineering issue, while the technical solution refers to the electronic component or structural design capable of addressing the engineering problem. It's worth noting that a single technical element may correspond to multiple technical solutions.
3. Incorporating game theoretic modeling into multiobjective decision making to efficiently determine the overall optimal product design solution.

The subsequent sections of this study are organized as follows: In Section “Literature Review and Research Framework”,

we analyzed relevant literature to develop the framework for this study. In section "Proposed Methodology", we present a product design model that converts user requirements into optimal technical solutions. This model integrates the Kano model, QFD theory, and an enhanced game theory model. To demonstrate the applicability of the design optimization model in this study, a separate case study is elaborated in Case study section. Conclusion and Discussion section summarizes the findings and addresses the limitations and future directions of the study.

## Literature review and research framework

### Analysis of the main steps of the KQGT model

Current research on user requirements driven design optimization models mostly addresses design method integration^11–13^. These studies have focused on two main areas: user needs analysis and the generation and selection of product technology solutions.

Most common user requirement analysis methods are based on the Kano model, which organizes and classifies attributes of user requirements by studying the relationship between user satisfaction and product features^7^. This model is now established in the field of product development and design and includes a complete theoretical analysis process; the Kano model has also been expanded by combination with demand weighting analysis. Haber et al.^14^ integrated AHP and the Kano model for analyzing user requirement weights to improve the reliability of user requirement system analysis. Sun et al.^15^ introduced the combined Kano-FAST method for college dormitory furniture design to construct a functional item prioritization model that captured important user requirements and translate them into technical elements. The aforementioned studies effectively assessed the weights of user requirements, but they included subjective decisions in the assessment process, resulting in large discrepancies between the weights assigned and the objective situation. For this reason, Lv et al.^16^ applied the Kano-AHP-QFD method for the design of innovative fruit logistics boxes, which reduced the subjectivity of user demand assignment by calibrating the weights based on user demand attributes. Therefore, this paper adopts the Kano model to analyze user demand attributes, introduces dual analysis to obtain the demand weights, and later calibrates the weights based on the demand attributes to enhance their accuracy.

The above analysis shows that the core issues in the generation of technical solutions and decision making are the establishment of high value technical elements and the decision making regarding the preference of technical solutions. Therefore, in this study, Functional Analysis System Techniques (FAST) be used to transform the user demand into technical elements. Quality Function Deployment (QFD) allows for the analysis of the importance of technical elements based on the weighting of user requirements and the autocorrelation of technical elements^17^. Therefore, this paper combines QFD theory with the results of user requirement analysis to establish high value technical elements. Subsequently, an expert group analyzes the high value technical elements and determines the corresponding technical solution, integrating the technical solution set containing multiple technical programs, this expert group comprised researchers with over five years of product design experience. In terms of program decision making, as the optimization of technical solutions has multi-objective attributes and the relationship between the objectives is one of dependency or conflict, a game theory model for studying the equilibrium solution under a conflict of interest can be introduced^18^, transforming the design solution preference problem into a multiobjective equilibrium game problem for fast technical solution preference^19^.

**Figure 1:**
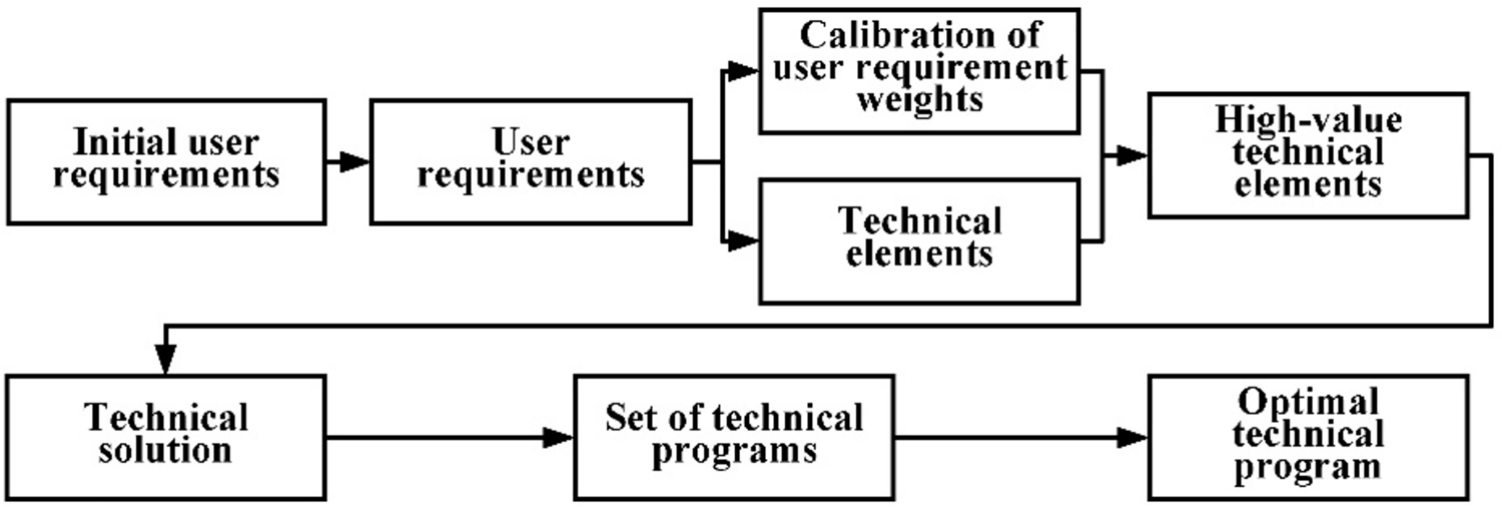
Main steps of the KQGT model.

In summary, this paper proposes a product optimization design model based on KQGT with the main steps shown in Fig. 1.

### KQGT model process planning

**Figure 2:**
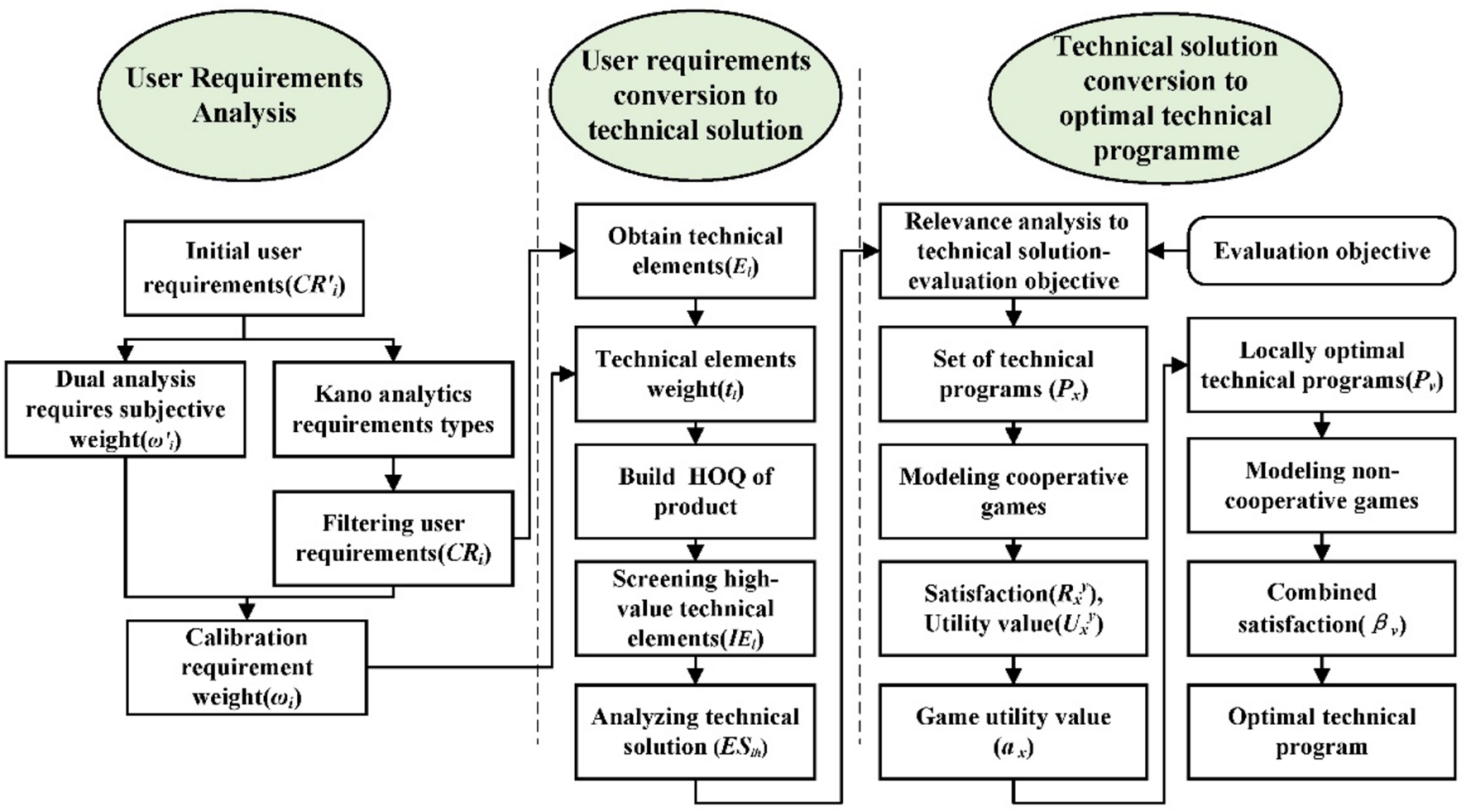
KQGT product design optimization model.

The KQGT product design optimization model flow is shown in Fig. 2 and is divided into three main parts:

1. User requirement mining: The Kano model is combined with the dual analysis method to classify the attributes, weights are assigned and the initial user requirements obtained from the preliminary research are calibrated, and the user requirements and weights that can be applied to the subsequent design are determined.
2. Transformation of user requirements into technical solutions: Because of the ambiguity of the user requirement descriptions, they are transformed into technical elements and evaluated in terms of value. The weights of the user requirements inform the construction of a product house of quality to derive high value technical elements and transform them into specific technical solutions.
3. Optimal technical solution decision making: The correlation between the design objectives and technical solutions is assessed, the technical solutions are combined to form a set of technical programs, and the cooperative–noncooperative tandem game model is employed to determine the final technical program.

**Figure 3:**
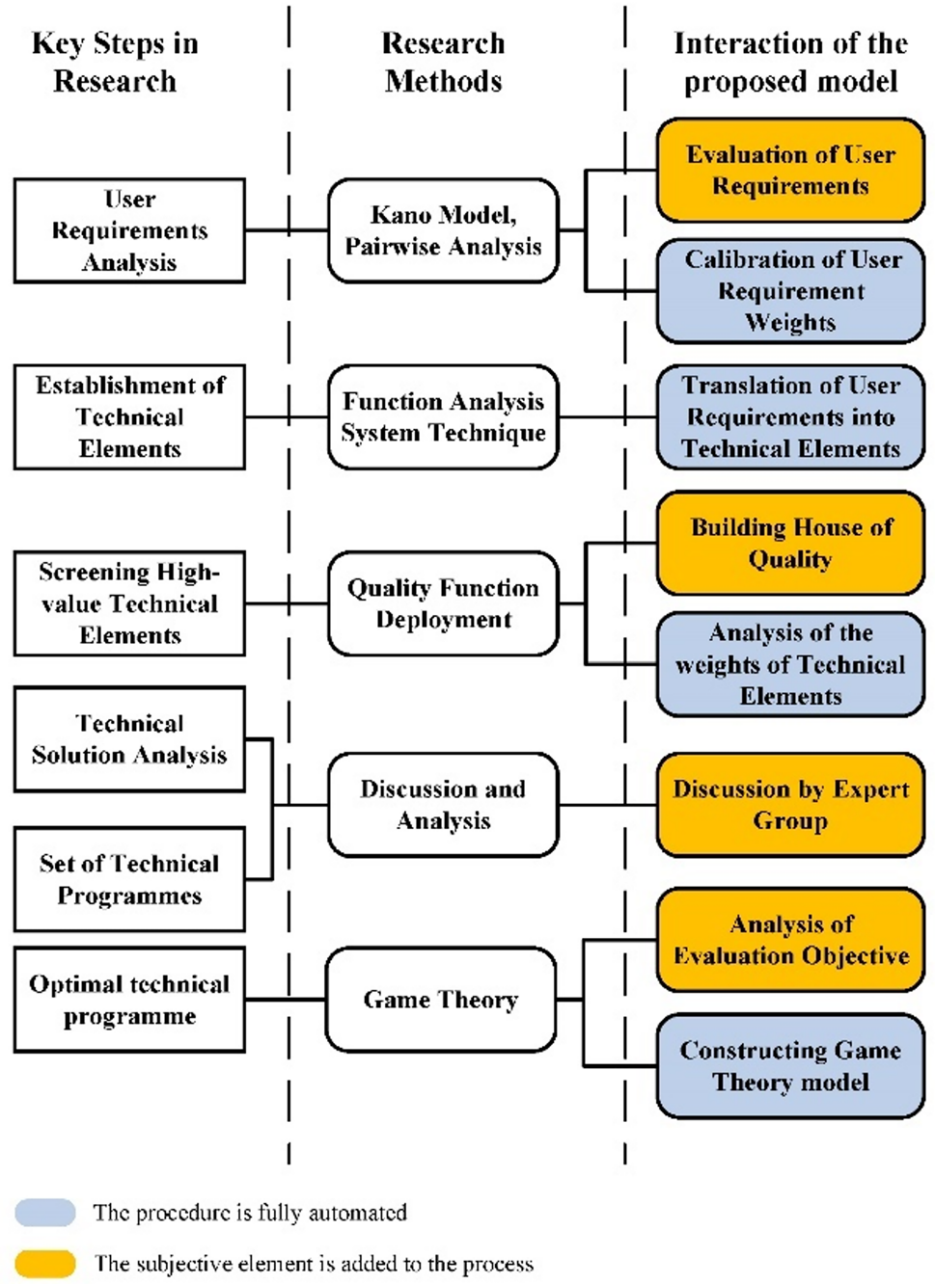
Research Methods of the KQGT model.

To enhance transparency regarding the researcher's interaction with the model and elucidate the impact of subjective factors, Fig. 3 illustrates the study's progression. The left portion delineates the purpose of each key research step, while the middle portion expounds on the theoretical framework or research methodology employed, the right portion side shows the subjective factors involved in each research methodology. Additionally, in the part of Interaction of the proposed model section distinguishes between objective theoretical approaches and subjective analyses, facilitating a clearer understanding of the researcher's engagement with the model.

## Proposed methodology

### User requirement analysis

User satisfaction with a product can be efficiently increased by tapping into the user requirements^4^. In order to explore multidimensional user requirements, comprehensive research based on offline interviews and web crawler collection is carried out to obtain and screen the initial user requirements; next, the Kano model is used to divide the demand attributes and remove non-differentiated demand and suspicious demand; finally, user requirement attributes are combined with dual analysis, and user requirement weights are assigned and calibrated to obtain scientific user requirement weights.

Traditionally, interviews or questionnaires are employed to establish user requirements, but the conversion efficiency and accuracy of such methods are low. Further research has focused on intelligent user requirement collection methods^20,21^, which have the advantages of speed, data coverage, accuracy, and reliability. In order to obtain more detailed and reliable data, a data collection approach combining web crawlers and offline interviews can be introduced to establish the initial user requirements. The details of this process are as follows:

A web crawler tool is used to collect information on the features and user requirements of the relevant products and transform them into text, and the Jieba tool in the Python 3 environment is used to organize the text and extract high-frequency requirement vocabularies^22^.

Offline interviews are conducted with the target group in the form of questionnaires and face to face interviews to determine their behavioral habits and preferences, and the results are organized into vocabularies of information on user expectations.

The high frequency requirement vocabularies collected by the web crawler and the user expectation vocabularies are organized using the KJ method to derive the initial user requirements (*CR*^*′*^_*i*_).

### User requirement processing based on Kano model

The qualitative segmentation of the user requirements is performed using the Kano model. According to the theory of the Kano model, five categories of user need attributes exist: Must be (M), One dimensional (O), Attractive (A), Indifferent (I), and Reverse (R). The research steps are as follows:

Based on the questionnaires designed and distributed in the preliminary research stage, initial user requirement questions are generated for both positive and negative dimensions. After collecting the questionnaires, each question attribute is divided according to Table 1^13^, and the results are eliminated for the Reverse (R) type.

**Table 1:** Initial user requirement Kano attribute categorization table for item *i*.

Initial User Requirement (*CR′*_*i*_)		Negative Question				
		Satisfactory	Desirable	Indifferent	Bearable	Unsatisfactory
Positive Question	Satisfactory	–	A	A	A	O
	Desirable	R	I	I	I	M
	Indifferent	R	I	I	I	M
	Bearable	R	I	I	I	M
	Unsatisfactory	R	R	R	R	–

To count the number of attributes of each initial user requirement, taking type M as an example, for a total of *m* respondents, if the *j*th respondent categorizes the* i*th user requirement as type M, then ${CM}_{ij}$ = 1; if it is categorized as another attribute type, then ${CM}_{ij}$ = 0. The scores for the different types of initial user requirement are computed using the following formulae:

1 $${M}_{i}=\sum_{j=1}^{m}{CM}_{ij}$$

2 $${O}_{i}=\sum_{j=1}^{m}{CO}_{ij}$$

3 $${A}_{i}=\sum_{j=1}^{m}{CA}_{ij}$$

4 $${I}_{i}=\sum_{j=1}^{m}{CI}_{ij}$$

Here:${M}_{i}$ is the score for the M-type categorization of initial user requirement *i*;${O}_{i}$ is the score for the O-type categorization of initial user requirement *i*;${A}_{i}$ is the score for the A-type categorization of initial user requirement *i*;${I}_{i}$ is the score for the I-type categorization of initial user requirement *i*.

Based on the type scores, the “better coefficient *B*_*i*_” and “worse coefficient *W*_*i*_” of the *i*th initial user requirement are calculated as follows:

5 $${B}_{i}=\frac{{A}_{i}+{O}_{i}}{{A}_{i}+{M}_{i}+{O}_{i}+{I}_{i}}$$

6 $${W}_{i}=\frac{{M}_{i}+{O}_{i}}{{A}_{i}+{M}_{i}+{O}_{i}+{I}_{i}}$$

**Figure 4:**
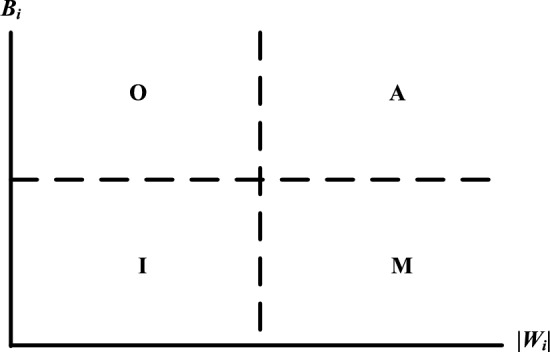
User requirement type division graph.

A user requirement type division graph is drawn, where the y-axis coordinate is ${B}_{i}$ and the x-axis coordinate is $|{W}_{i}|$, and an attribute quartile graph with median values ${B}_{i}$ and $|{W}_{i}|$ is established as a reference to clarify the requirement attributes. The attribute division is shown in Fig. 4. The I-type requirements are removed, and the remaining needs are counted as valid user needs, *CR*_*i*_. The importance of the valid requirement attributes is ranked as follows: M > O > A^15^.

### Assignment and calibration of user requirement weights

To make up for the shortcomings of the Kano model in quantitative analysis, a comparative judgment matrix based on Pairwise Analysis was introduced to analyze the weight of each user requirements. A 9 level scale is used to compare the user requirements and construct the judgment matrix ***M***^23^ in order to derive subjective weight values for each user requirement (*ω′*_*i*_). To enhance the credibility of the weight calculations, a combination of qualitative and quantitative methods is employed^24^. The subjective weight value (*ω′*_*i*_) is calibrated in conjunction with the user demand attributes to derive the weight value (*ω*_*i*_). The process is divided into two steps:

1. Subjective weight calculation: In this paper, the geometric mean method is used to solve the weight vector in the comparison judgment matrix. The formula is as follows^25^:
  7 $${M}_{a}=\prod_{a=1}^{n}{W}_{ab}$$
  8 $${w}_{a}=\sqrt[n]{{M}_{a}}$$
  9 $${\omega {\prime}}_{i}=\frac{{w}_{a}}{{\sum }_{a=1}^{n}{w}_{a}}$$

Here:${W}_{ab}$ is an element in row a, column b in the judgment matrix;$n$ is the order number of the judgment matrix.

**Table 2:** RI values.

$n$	3	4	5	6	7	8	9	10	11	12	13	14
*RI*	0.52	0.89	1.12	1.26	1.36	1.41	1.46	1.49	1.52	1.54	1.56	1.58

**Table 3:** Values of k for each type of requirement.

User Requirement Type	M	O	A
*k*	*k*_M_ = 2	*k*_O_ = 1	*k*_A_ = 0.5

(2) Consistency test: To ensure the validity of the subjective weight assignment, Saaty’s probabilistic consistency test is used^23^. The consistency of the judgment matrix is calculated by solving for its largest eigenvalue ${\lambda }_{max}$. A result of $CR$ < 0.1 is considered to pass the consistency test, and the subjective weight value ${\omega {\prime}}_{i}$ is available. Otherwise, the judgment matrix needs to be adjusted until it passes the consistency test^26^. The formula for the consistency test is as follows, where the value of *RI* is shown in Table 2 and depends on the matrix order *n*:In this formula:$CI$ is the consistency index of the judgment matrix;${\lambda }_{max}$ is the maximum eigenvalue of the judgment matrix;$CR$ is the coherence ratio factor;$RI$ is the average stochastic consistency indicator.
  10 $$CI=\frac{{\lambda }_{max}-n}{n-1}$$
  11 $$CR=\frac{CI}{RI}$$

(3) Calibration of user requirement weights: The weights are corrected by setting the corresponding correction value *k* according to the importance of the user requirement type and normalized as user requirement weights (*ω*_*i*_). Table 3 lists the corrected values of *k* for each type of requirement.

### Translation of user requirements into technical elements

**Figure 5:**
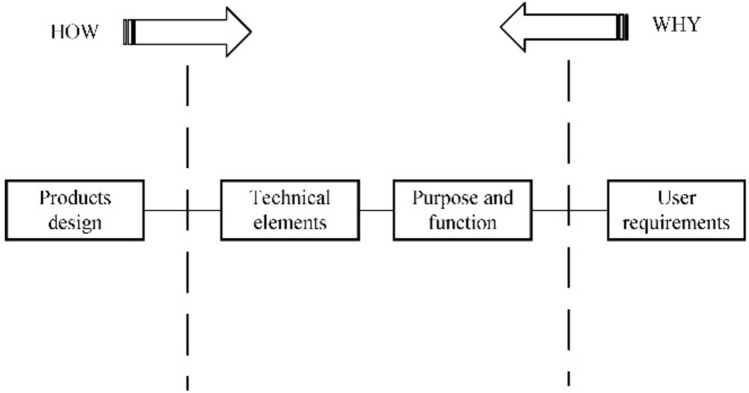
Functional analysis system techniques.

This study addresses the common challenge of ambiguous user requirements by employing Functional Analysis System Techniques (FAST)^15^. FAST enables a systematic exploration of user requirements, allowing for a clearer delineation and comprehension of their purpose and function. Subsequently, this method facilitates the derivation of precise engineering specifications, denoted as technical elements (*E*_*l*_). The structure of the FAST model, illustrated in Figure 5, exemplifies the systematic approach employed in this process.

### HOQ-based screening of high-value technical elements

**Figure 6:**
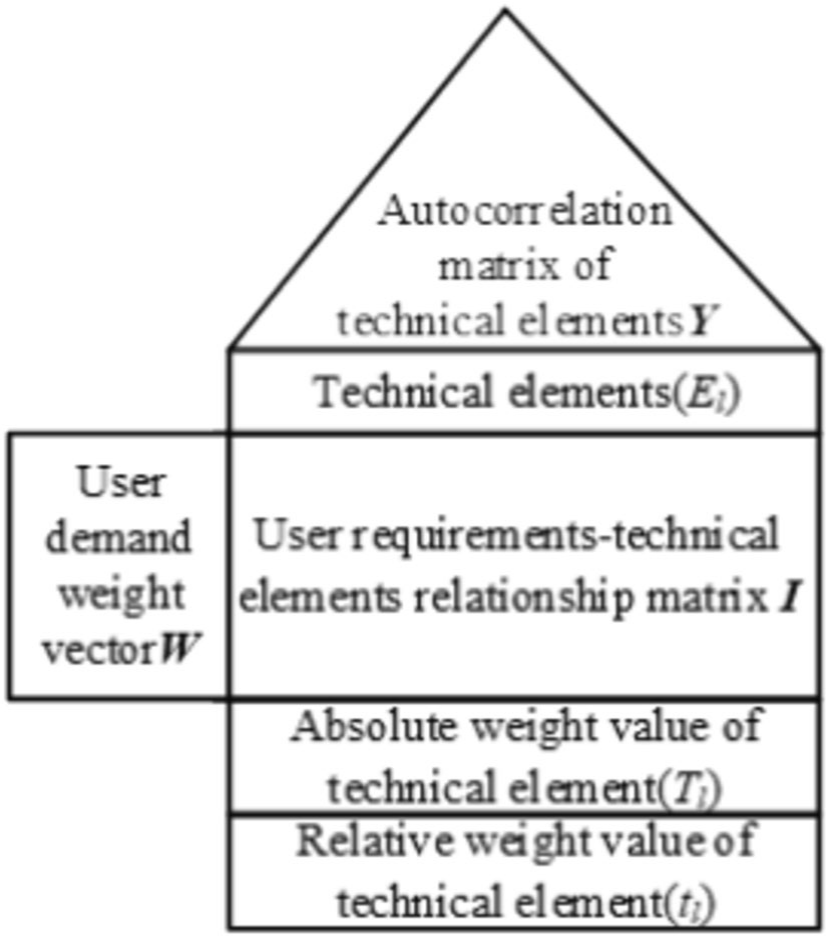
The HOQ of product technical elements.

The role of a house of quality (HOQ) is mainly to establish the correlation between user requirements and technical elements and clarify the focus in product design through layer by layer transformation so that the subsequent design can be carried out in a targeted manner. The construction of the product HOQ technical elements is shown in Fig. 6, where the left wall is the user demand weight vector ***W***, as in Formula (12). The roof is the autocorrelation matrix of technical elements ***Y***, where “none, ☆, △, □, ○” correspond to “uncorrelated, weakly correlated, moderately correlated, strongly correlated, autocorrelated” and the values “0, 1, 3, 5, 9”, respectively. The ceiling corresponds to technical elements (*E*_*l*_). The room represents the user requirements–technical elements relationship matrix ***I,*** where the correlation scores are based on a 9 point scale and ${I}_{il}$ denotes the relevance of the *i*th user requirement to the *l*th technology element. The floor is the technical element absolute weight (T_*l*_) and relative weight (*t*_*l*_).

The weights of the technical elements (*E*_*l*_) are calculated as follows ^27,28^:

12 $$W = \omega_{i} ,{ }i = 1,2, \ldots ,n$$

13 $$T={W}^{T}IY$$

14 $$T=\left({T}_{l}\right), l=\text{1,2}, \dots , m$$

15 $${t}_{l}=\frac{{T}_{l}}{\sum_{i=1}^{n}{T}_{l}}$$

In this formula:$T$ is the vector of absolute weight values for the technical elements;${T}_{l}$ is the absolute weight value of technical element *l*;${t}_{l}$ is the relative weight value of technical element *l*.

To further clarify the design focus, high-value technical elements (*IE*_*l*_) are established using the relative weight values of the technical elements (*t*_*l*_) as a reference. According to the ABC classification method, factors with a weight proportion above 95% have a larger impact on a project^29^, so the relative weight values of the technical elements (*t*_*l*_) are arranged from large to small and added together until ${t}_{l}$ reaches 0.95. The technical elements (*E*_*l*_) included in this sum are recorded as the high-value technical elements (*IE*_*l*_), and the residual technical elements are eliminated. The high-value technical elements are the engineering objectives to be met by the product technical program. The expert group conducts a technical assessment based on the high-value technical elements (*IE*_*l*_), determines the technical solution (*ES*_*lh*_), and analyzes its feasibility. (*ES*_*lh*_) is the *h*th technical solution of the *l*th high-value technical element. The technical solutions (*ES*_*lh*_) are combined to generate a set of multiple technical programs (*P*_*x*_). The complete set of technical programs obtained is organized to prepare for optimal solution decision-making in subsequent studies.

### Game theory research

Game theory is a theoretical tool to study how two or more interacting game parties conduct equilibrium solution under the environment of conflict of interest. And there is also a multi-objective equilibrium problem in product design, and there is a conflict or dependence between the objectives.

Game theory is mainly composed of three basic elements: game party, strategy and utility. Game theory models are categorized into cooperative and non-cooperative game models according to whether a binding agreement can be reached. Cooperative games prioritize collective rationality to enhance overall interests, aiming for a solution that maximizes overall benefit. Non-cooperative games, on the other hand, emphasize individual rationality in strategic decision-making, seeking equilibrium solutions advantageous to each player.

### Decision making applications of game theory models

In this study, the main problem faced in the design decision-making stage is the coupling and mutual influence of the technical solutions in the technical programs set, and the advantages and disadvantages of different technical solutions are difficult to be formally expressed and analyzed. Thus, this issue is reframed as a multi-objective game problem, wherein parallel decision-making occurs while acknowledging the interrelated nature of multiple objectives. The objective is to attain an optimal product solution that maximally considers the high-value technical elements.

Hence, the decision-making steps of the design scheme based on game theory in this study are as follows:

1. Correlation analysis.

Based on the principle of parallel design, the expert group analyzes and discusses the evaluation objectives in the optimization stage of the design solution. Each technical solution (*ES*_*lh*_) is clustered together with an evaluation objective^30^, and the technical solution–evaluation objective relevance scoring is carried out, adopting a 5-level scale. The relevance value (*r*_*lh*_) indicates the relevance of the technical solution (*ES*_*lh*_) to the corresponding evaluation objective, as shown in Table 4.

(2) Constructing Game Theory model. The correlation based analysis examines the coupling relationship among technical solutions to screen the ensemble. Here, the evaluation objective serves as the game party, the technical solution as the game party strategy, and the utility function of the technical solution ensemble is derived from the correlation value for the game utility value. In summary, calculate the satisfaction level of each technical program based on Table 4, and the calculation process is shown in formula (16)–(17), derive the satisfaction value (*R*_*x*_) of each game party. Since there is a mutual influence relationship between the game parties in the decision-making process, the utility value of each game party is calculated based on the satisfaction of the game parties with the strategies of each game party, and the calculation process is shown in formula (18).
  16 $$T\left({r}_{l}^{y}\right)=\left\{\begin{array}{c}0\\ \frac{{r}_{lh}^{y}-1}{5-1}\end{array}\right.\genfrac{}{}{0pt}{}{(r=1)}{(1<{r}_{lh}^{y}<5)}$$
  17 $${R}_{x}^{y}=\frac{1}{e}\sum_{l=1}^{e}T\left({r}_{l}^{y}\right)$$
  18 $$\left\{\begin{array}{c}{U}_{1}^{1}=\frac{{R}_{1}^{1}}{{R}_{1}^{2}+{R}_{1}^{3}+\dots +{R}_{1}^{y}}\\ \vdots \\ {U}_{x}^{y}=\frac{{R}_{x}^{y}}{{R}_{x}^{1}+{R}_{x}^{1}+\dots +{R}_{x}^{y-1}}\end{array}\right.$$

In this formula:${r}_{lh}^{y}$ is the mean value of the correlation between the *h*th technical solution in the *l*th technical element and the* y*th evaluation objective;$T\left({r}_{l}^{x}\right)$ is the extent to which the *l*th technical element meets the *x*th evaluation objective;$e$ is the number of technical elements for the *x*th evaluation objective;${R}_{x}^{y}$ is the satisfaction of the *y*th evaluation objective with program *x*;${U}_{x}^{y}$ is the utility value of program *x* for the *y*th evaluation objective;

**Table 4:** Technical solution–evaluation objective correlation analysis.

Evaluation Objective	High-Value Technical Elements (*IE*_*l*_)	Technical Solution (*ES*_*lh*_) |Relevance Value (*r*_*lh*_)
Objective 1	$I{E}_{1}$	*ES*_11_|*r*_11_, *ES*_12_|*r*_12_, …, *ES*_1*h*_|*r*_1*h*_
	$I{E}_{2}$	*ES*_21_|*r*_21_, *ES*_22_|*r*_22_, …, *ES*_2*h*_|*r*_2*h*_
Objective 2	$I{E}_{3}$	*ES*_31_|*r*_31_, *ES*_32_|*r*_32_, …, *ES*_3*h*_|*r*_3*h*_

(3) Non-cooperative games seek locally optimal technical programs. To ensure unbiased outcomes in the later stages of the game process, it's essential to prioritize individual revenue maximization for each game party. Therefore, we initially employ a non-cooperative game approach for screening, establishing utility constraints to ensure the reliability of subsequent program decision-making. The calculation process is shown in formula (19). The arithmetic mean of the maximum and minimum values of the game utility value is counted as the formulation of the screening threshold^30^. Technical programs with utility values surpassing this threshold are deemed the set of locally technical programs. (*P*_*v*_).In this formula:${\alpha }_{x}$ is the game utility value of program *Px*.
  19 $${\alpha }_{x}={U}_{x}^{1}*{U}_{x}^{2}*\cdots *{U}_{x}^{y}$$

(4) Cooperative games seek overall optimal technical programs. The technical programs set identified through the non-cooperative game represents the local optimal technical programs, requiring further refinement via cooperative game theory to attain the overall optimal technical programs. This entails seeking the optimal technical programs with maximum overall benefit. The calculation process is shown in formula (20).In this formula:${\beta }_{v}$ is the overall satisfaction of the evaluation objective by technical program *v*;${i}_{1}{, i}_{2}$ are the numbers of gaming parties;*U*_*m*_(*S*) is the utility value of the high-merit indicator game party;*U*_*m-min*_(*S*) is the minimum utility value of the high-merit indicator game party;*U*_*n*_(*S*) is the utility value of the low-merit indicator game party;*U*_*n-max*_(*S*) is the maximum utility value of the low-merit indicator game party.
  20 $${\beta }_{v}=\prod_{m=1}^{{i}_{1}}\left[{U}_{m}\left(S\right)-{U}_{m-min}\left(S\right)\right]*\prod_{n=1}^{{i}_{2}}\left[{U}_{n-max}\left(S\right)-{U}_{\text{n}}\left(S\right)\right]$$

The game parties are categorized by the expert group as either high or low merit indicators based on the evaluation objective. For example, the structural or technological game parties are high-merit indicators, and the economic game parties are low-merit indicators. Therefore, the technical program with the highest overall satisfaction (*β*_*v*_) is selected as the optimal technical programs.

## Case study

This study found that most medicinal bath water heaters cannot achieve high user satisfaction because the user requirements were not transformed into reliable and effective technical elements to guide the early stage of the design process and the analysis of the user requirements was vague. Therefore, the KQGT model was adopted for the product design of a medicinal bath water heater as an example. This study invited ten scholars from the Industrial Design Institute of Shaanxi University of Science and Technology, each possessing over five years of product development experience, to form an expert group for this study, the expert panel was established to facilitate the subjective analysis component of this study.

### User requirement and type analyses

The experimental research of this study was approved by the Academic Committee of the College of Design and Art, Shaanxi University of Science and Technology and the Institute of Industrial Design, Shaanxi University of Science and Technology. The whole research process was conducted under their supervision. Before the experiment, all subjects were informed of the experimental process and precautions, and the consent of each subject or his/her legal guardian was obtained.

Offline interviews were conducted with practitioners in Chinese medicinal bathrelated industries to obtain information on users’ expectations of the product. Afterward, web crawler tools were used to collect information on the relevant functions of similar medicinal bath water heaters, which was transformed into text, and the initial user requirements (*CR′*_*i*_) were obtained after processing, as shown in Table 5.

**Table 5:** Initial user requirements of medicinal bath water heater.

Initial User Requirement (*CR′*_*i*_)	Requirement Vocabulary	Initial User Requirement (*CR′*_*i*_)	Requirement Vocabulary
*CR’*_1_	Boil medicine	*CR′*_9_	Homoiothermy bath
*CR′*_2_	Prevent dry burning	*CR′*_10_	Water temperature adjustment
*CR′*_3_	Anti-scalding	*CR′*_11_	Solid–liquid separation
*CR′*_4_	Fault self-checking	*CR′*_12_	Small volume
*CR′*_5_	Self-cleaning	*CR′*_13_	Easy disassembly
*CR′*_6_	Filtration	*CR′*_14_	Touchscreen control
*CR′*_7_	End reminder	*CR′*_15_	Unmute
CR′8	Start reminder		

**Figure 7:**
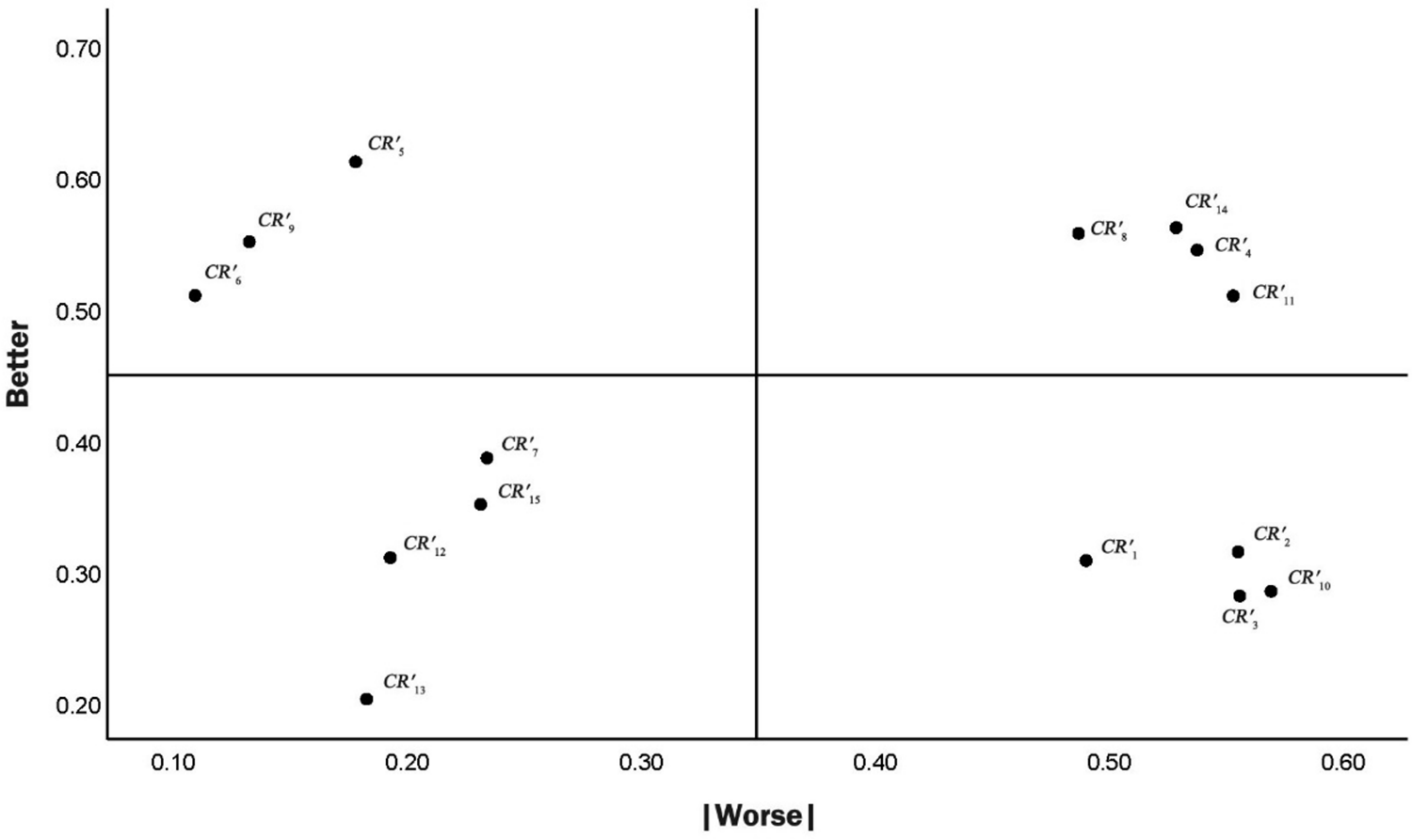
Quartile diagram of initial user requirements.

A questionnaire was designed according to the initial user requirements and the design principles of the Kano questionnaire in Table 2. A total of 180 Chinese herbal bath users and practitioners in Chinese medicinal bathrelated industries were selected to complete the questionnaire, and 165 valid questionnaires were ultimately obtained. The attribute categorization matrix in Table 1 and Eqs. (1)–(6) were employed to draw the quartile diagram of user requirements in Fig. 7. Based on this diagram, we divided and screened the requirements, and the remaining items were included as user requirements (*CR*_*i*_), see Table 6.

**Table 6:** Segmentation of user requirement types.

Type of Requirement	User Requirement (*CR*_*i*_)	Type of Requirement	User Requirement (*CR*_*i*_)
M	Boil medicine (*CR*_1_)	A	Self-cleaning (*CR*_5_)
	Prevent dry burning (*CR*_2_)		Homoiothermy bath (*CR*_6_)
	Anti-scalding (*CR*_3_)		Filtration (*CR*_8_)
	Water temperature adjustment (*CR*_9_)		Touchscreen control (*CR*_11_)
O	Fault self-checking (*CR*_4_)		
	Start reminder (*CR*_7_)		
	Solid–liquid separation (*CR*_10_)		

### User requirements weighting analysis and calibration

We invited 10 practitioners in the field of industrial design to score the importance of user needs and constructed judgment matrix M. According to Eqs. (7)–(11), *λ*_*max*_ = 11.91 and *CI* = 0.092, because *n* = 11 in the considered judgment matrix. Table 2 shows that at this time, *RI* = 1.52, and we calculated that *CR* = 0.06; therefore, the judgment matrix passed the consistency test. The subjective weight value (*ω′*_*i*_) of each user requirement is shown in Table 7.

**Table 7:** Weighting correction table.

Type of Requirement	Revised Value (*k*)	User Requirement (*CR*_*i*_)	Subjective Weight (*ω′*_*i*_)	Weight(*ω*_*i*_)
M	*k*_M_ = 2	*CR*_1_	0.13	0.17
		*CR*_2_	0.16	0.20
		*CR*_3_	0.17	0.21
		*CR*_9_	0.21	0.27
O	*k*_O_ = 1	*CR*_4_	0.05	0.03
		*CR*_7_	0.02	0.01
		*CR*_10_	0.06	0.04
A	*kA* = 0.5	*CR*_5_	0.04	0.01
		*CR*_6_	0.07	0.02
		*CR*_8_	0.08	0.03
		*CR*_11_	0.01	0.01

$$M\hspace{0.17em}=\hspace{0.17em}\left[\begin{array}{ccccccccccc}1.00& 0.30& 0.89& 4.33& 3.33& 3.45& 6.67& 2.33& 0.33& 4.00& 8.00\\ 2.56& 1.00& 1.00& 3.03& 5.00& 3.45& 5.56& 2.00& 1.00& 4.55& 7.69\\ 1.12& 1.00& 1.00& 4.17& 5.88& 3.33& 6.25& 3.03& 1.00& 4.76& 7.69\\ 0.23& 0.33& 0.24& 1.00& 2.00& 0.43& 4.17& 0.43& 0.15& 1.00& 6.25\\ 0.30& 0.20& 0.17& 0.50& 1.00& 0.50& 3.13& 0.25& 0.16& 0.27& 6.25\\ 0.29& 0.29& 0.30& 2.33& 2.00& 1.00& 4.33& 1.44& 0.20& 1.50& 6.00\\ 0.15& 0.18& 0.16& 0.24& 0.32& 0.23& 1.00& 0.16& 0.14& 0.23& 3.03\\ 0.43& 0.50& 0.33& 2.33& 4.00& 0.69& 6.33& 1.00& 0.23& 1.00& 6.67\\ 3.00& 1.00& 1.00& 6.67& 6.33& 5.00& 7.00& 4.33& 1.00& 5.33& 8.00\\ 0.25& 0.22& 0.21& 1.00& 3.67& 0.67& 4.33& 1.00& 0.19& 1.00& 6.00\\ 0.13& 0.13& 0.13& 0.16& 0.16& 0.17& 0.33& 0.15& 0.13& 0.17& 1.00\end{array}\right]$$

The subjective weight value of each user requirement (*ω′*_*i*_) was corrected according to the corrected value of each requirement type in Table 3, and the user requirement weight value (*ω*_*i*_) was derived from the normalization process after correction, which is also shown in Table 7.

### Translating user requirements into technical solutions

**Figure 8:**
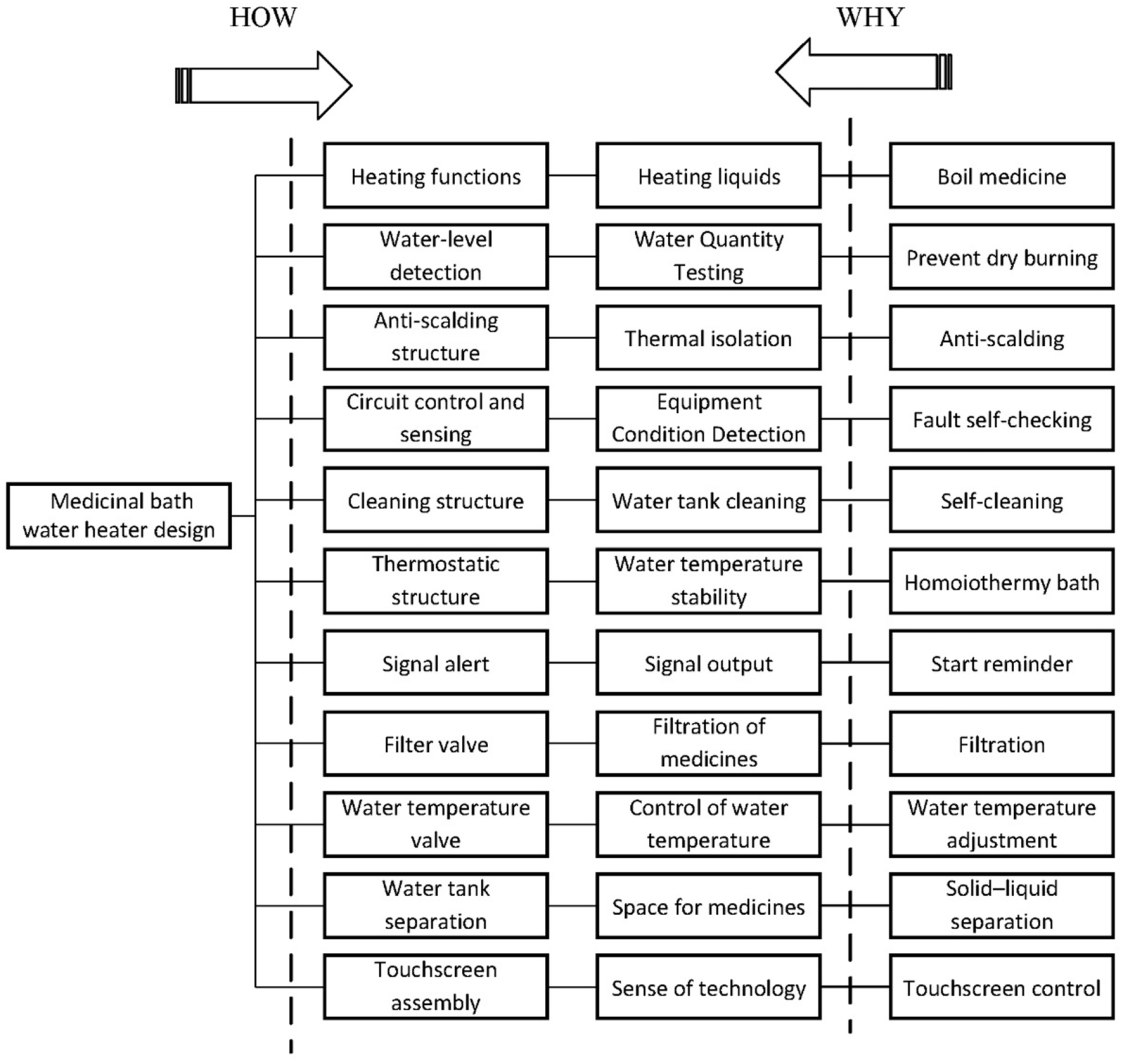
User requirement–Technical element transformation.

Based on Table 6, we utilize the FAST theory to convert user requirements (*CR*_*i*_) into technical elements (*E*_*l*_), as demonstrated in Fig. 8.

In order to facilitate subsequent research, the technical elements derived from each user requirement were systematically organized and coded, as outlined in Table 8.

**Table 8:** User requirements–Technical elements.

User Requirement (*CR*_*i*_)	Technical Solution (*E*_*l*_)	User Requirement (*CR*_*i*_)	Technical Solution (*E*_*l*_)
*CR*_1_	Heating functions (*E*_1_)	*CR*_7_	Signal alert (*E*_7_)
*CR*_2_	Water-level detection (*E*_2_)	*CR*_8_	Filter valve (*E*_8_)
*CR*_3_	Anti-scalding structure (*E*_3_)	*CR*_9_	Water temperature valve (*E*_9_)
*CR*_4_	Circuit control and sensing (*E*_4_)	*CR*_10_	Water tank separation (*E*_10_)
*CR*_5_	Cleaning structure (*E*_5_)	*CR*_11_	Touchscreen assembly (*E*_11_)
*CR6*	Thermostatic structure (*E6*)		

### HOQ of medicinal bath water heater

**Figure 9:**
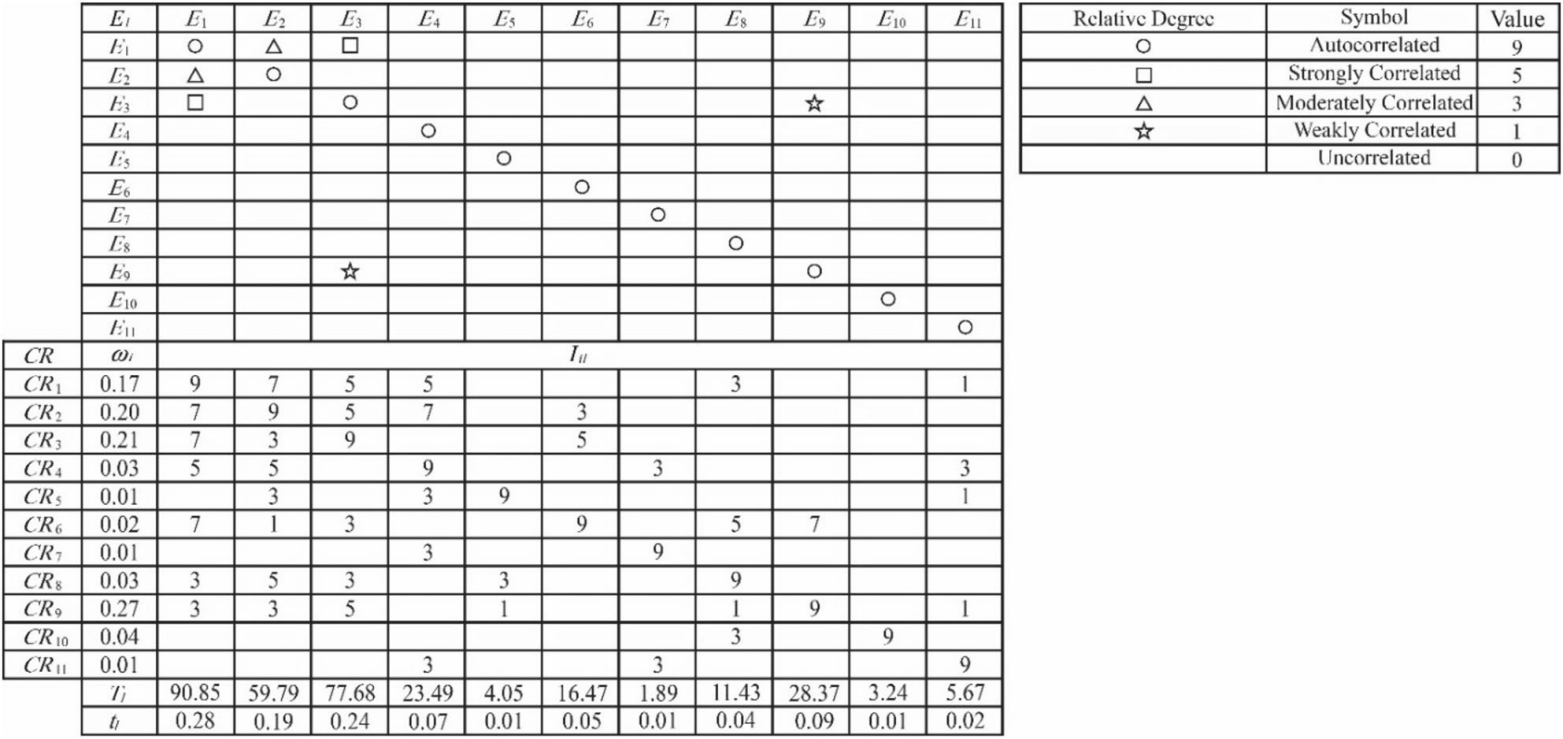
HOQ of medicinal bath water heater.

Five technical experts involved in the design of the medicinal bath water heater were invited to form an expert group that would quantitatively evaluate the correlation between various user requirements and technical elements; calculate the absolute and relative weights of each technical element according to Eqs. (12)–(15); and construct a house of quality for the technical elements of the medicinal bath water heater, as shown in Fig. 9.

The technical element autocorrelation matrix ***Y***, the user requirement–technical element relationship matrix ***I***, and the user requirement weight vector ***W*** were as follows:

$$Y\hspace{0.17em}=\hspace{0.17em}\left[\begin{array}{ccccccccccc}9& 3& 5& 0& 0& 0& 0& 0& 0& 0& 0\\ 3& 9& 0& 0& 0& 0& 0& 0& 0& 0& 0\\ 5& 0& 9& 0& 0& 0& 0& 0& 1& 0& 0\\ 0& 0& 0& 9& 0& 0& 0& 0& 0& 0& 0\\ 0& 0& 0& 0& 9& 0& 0& 0& 0& 0& 0\\ 0& 0& 0& 0& 0& 9& 0& 0& 0& 0& 0\\ 0& 0& 0& 0& 0& 0& 9& 0& 0& 0& 0\\ 0& 0& 0& 0& 0& 0& 0& 9& 0& 0& 0\\ 0& 0& 1& 0& 0& 0& 0& 0& 9& 0& 0\\ 0& 0& 0& 0& 0& 0& 0& 0& 0& 9& 0\\ 0& 0& 0& 0& 0& 0& 0& 0& 0& 0& 9\end{array}\right]$$

$$I\hspace{0.17em}=\hspace{0.17em}\left[\begin{array}{ccccccccccc}9& 7& 5& 5& 0& 0& 0& 3& 0& 0& 1\\ 7& 9& 5& 7& 0& 3& 0& 0& 0& 0& 0\\ 7& 3& 9& 0& 0& 5& 0& 0& 0& 0& 0\\ 5& 5& 0& 9& 0& 0& 3& 0& 0& 0& 3\\ 0& 3& 0& 3& 9& 0& 0& 0& 0& 0& 1\\ 7& 1& 3& 0& 0& 9& 0& 5& 7& 0& 0\\ 0& 0& 0& 3& 0& 0& 9& 0& 0& 0& 0\\ 3& 5& 3& 0& 3& 0& 0& 9& 0& 0& 0\\ 3& 3& 5& 0& 1& 0& 0& 1& 9& 0& 1\\ 0& 0& 0& 0& 0& 0& 0& 3& 0& 9& 0\\ 0& 0& 0& 3& 0& 0& 3& 0& 0& 0& 9\end{array}\right]$$

$$W\hspace{0.17em}=\hspace{0.17em}{\left[\begin{array}{ccccccccccc}0.17& 0.20& 0.21& 0.03& 0.01& 0.02& 0.01& 0.03& 0.27& 0.04& 0.01\end{array}\right]}^{T}$$

$$T\hspace{0.17em}=\hspace{0.17em}\left[\begin{array}{ccccccccccc}90.85& 59.79& 77.68& 23.49& 4.05& 16.47& 1.89& 11.43& 28.37& 3.24& 5.67\end{array}\right]$$

After removing the technical elements whose relative weights were below the threshold value, seven high-value technical elements (*IE*_*l*_) were included, and the expert group analyzed the feasible technical solutions ${ES}_{lh}$ accordingly, as shown in Table 9.

**Table 9:** High-value technical elements—technical solution analysis.

High-Value Technical Element ($I{E}_{l}$)	Technical Solutions (${ES}_{lh}$)
Heating functions (*IE*_*1*_)	Built in U-shaped heating rod (*ES*_11_)
	External heating plate (*ES*_12_)
Water-level detection (*IE*_*2*_)	Submerged liquid level sensor (*ES*_21_)
	Electric capacity type liquidometer (*ES*_22_)
Anti-scalding structure (*IE*_*3*_)	Polystyrene insulation layer (*ES*_31_)
	Rock wool insulation layer (*ES*_32_)
Circuit control and sensing (*IE*_*4*_)	Single chip microcomputer control (*ES*_41_)
Thermostatic structure (*IE*_*5*_)	Electronic temperature control valve (*ES*_51_)
	Thermosensitive thermostatic valve (*ES*_52_)
Filter valve (*IE*_*6*_)	Pipe connection filter valve (*ES*_61_)
	Water tank built in filter device (*ES*_62_)
Water temperature valve (*IE*_*7*_)	External water temperature valve (*ES*_71_)
	Constant temperature regulating valve (*ES*_72_)

### Technical solution cluster correlation analysis

In order to improve the efficiency of medicinal bath water heater product development, the parallel design concept was used for solution optimization^19^, based on which the evaluation objectives were derived, namely cost (c), quality (q), and operation (o).

The feasibility of the technical solution was analyzed with reference to the evaluation objectives. Regarding the anti-scalding structure design, the rock wool insulation layer (*ES*_32_) was deemed to have a short lifespan, so the technical solution *ES*_32_ was removed. Regarding the filtration structure, a built in filtration device in the water tank (*ES*_62_) presented operational safety risks, so the technical solution *ES*_62_ was removed. At the same time, the expert group was invited to conduct cluster analysis and correlation scoring for each technical solution based on the evaluation objectives, and the results are shown in Table 10.

**Table 10:** Technical solution cluster correlation analysis.

Evaluation Objective	High-Value Technical Element ($I{E}_{l}$)	Technical Solution (${ES}_{lh}$)|Relevance Value (*r*_*lh*_)	
Cost (c)	$I{E}_{2}$	*ES*_21_|4.70	*ES*_22_|3.80
	$I{E}_{5}$	*ES*_51_|4.20	*ES*_52_|3.50
	$I{E}_{7}$	*ES*_71_|4.50	*ES*_72_|3.90
Quality (q)	$I{E}_{1}$	*ES*_11_|4.2	*ES*_12_|4.0
	$I{E}_{3}$	*ES*_31_|4.0	
	$I{E}_{6}$	*ES*_61_|4.2	
Operation (o)	$I{E}_{4}$	*ES*_41_|3.9	

Based on the selection of components, if the electronic temperature control valve *ES*_51_ was used in the thermostatic structure, the water temperature valve could only be adjusted through the external water temperature control valve *ES*_71_; conversely, if technical solution *ES*_52_ was used, then technical solution *ES*_72_ had to be used. Based on this, a total of eight (2 * 2 * 2 * 1 * 1 * 1) technical programs were identified for the technical solution *P*_*x*_, as shown in Table 11.

**Table 11:** The set of technical programs.

Technical Program (*P*_*x*_)	Set of Technical Solutions
*P*_1_	*ES*_11_, *ES*_21_, *ES*_31_, *ES*_41_, *ES*_51_, *ES*_61_, *ES*_71_
*P*_2_	*ES*_12_, *ES*_21_, *ES*_31_, *ES*_41_, *ES*_51_, *ES*_61_, *ES*_71_
*P*_3_	*ES*_11_, *ES*_22_, *ES*_31_, *ES*_41_, *ES*_51_, *ES*_61_, *ES*_71_
*P*_4_	*ES*_12_, *ES*_22_, *ES*_31_, *ES*_41_, *ES*_51_, *ES*_61_, *ES*_71_
*P*_5_	*ES*_11_, *ES*_21_, *ES*_31_, *ES*_41_, *ES*_52_, *ES*_61_, *ES*_72_
*P*_6_	*ES*_12_, *ES*_21_, *ES*_31_, *ES*_41_, *ES*_52_, *ES*_61_, *ES*_72_
*P*_7_	*ES*_11_, *ES*_22_, *ES*_31_, *ES*_41_, *ES*_52_, *ES*_61_, *ES*_72_
*P*_8_	*ES*_12_, *ES*_22_, *ES*_31_, *ES*_41_, *ES*_52_, *ES*_61_, *ES*_72_

### Optimal technical program selection

Combining the contents of Tables 8 and 9, the satisfaction (${R}_{x}^{c}$, ${R}_{x}^{q}$, ${R}_{x}^{o}$) and utility (${U}_{x}^{c}$, ${U}_{x}^{q}$, ${U}_{x}^{o}$) values of each game party were determined according to Eqs. (16)–(19), and the results are shown in Table 12.

**Table 12:** The utility matrix of the non-cooperative game of technical programs.

*P*_*x*_	${R}_{x}^{c}$	${R}_{x}^{q}$	${R}_{x}^{o}$	${U}_{x}^{c}$	${U}_{x}^{q}$	${U}_{x}^{o}$	${\alpha }_{x}$
*P*_1_	0.8667	0.7916	0.7250	0.5715	0.4975	0.4370	0.1243
*P*_2_	0.8667	0.7750	0.7250	0.5780	0.4868	0.4415	0.1242
*P*_3_	0.7916	0.7916	0.7250	0.5221	0.5221	0.4577	0.1248
*P*_4_	0.7916	0.7750	0.7250	0.5280	0.5109	0.4627	0.1248
*P*_5_	0.7583	0.7916	0.7250	0.4997	0.5341	0.4677	0.1248
*P*_6_	0.7583	0.7750	0.7250	0.5053	0.5226	0.4729	0.1249
*P*_7_	0.6833	0.7916	0.7250	0.4502	0.5625	0.4915	0.1245
*P*_8_	0.6833	0.7750	0.7250	0.4553	0.5504	0.4973	0.1246

The arithmetic mean of the maximum and minimum values of ${\alpha }_{x}$ is included as a screening threshold, and the set of locally optimal technical programs (*P*_*v*_) was filtered to include P3, P4, P5, P6, and P8.

### Optimal technical program

According to Eq. (20), the combined satisfaction (${\beta }_{v}$) of each design solution was determined, and the solution with the highest satisfaction was identified as the optimal technical program. The results are shown in Table 13.

**Table 13:** Technical program cooperation game matrix.

*P*_*v*_	Utility Value of Game Party			${\beta }_{v}$/%
	${U}_{x}^{c}$	${U}_{x}^{q}$	${U}_{x}^{o}$	
*P*_3_	0.5221	0.5221	0.4577	0.0000
*P*_4_	0.5280	0.5109	0.4627	0.0000
*P*_5_	0.4997	0.5341	0.4677	0.0010
*P*_6_	0.5053	0.5226	0.4729	0.0008
*P*_8_	0.4553	0.5504	0.4973	0.0000

Table 13 indicates that the combined satisfaction of *P*_5_ was the highest, and this was designated as the optimal technical program. The specific technical solutions are shown in Table 14.

**Table 14:** Optimal technical program for medicinal bath water heater.

High-Value Technical Elements (*IE*_*l*_)	Technical Solution (*ES*_*lh*_)	High-Value Technical Elements (*IE*_*l*_)	Technical Solution (*ES*_*lh*_)
*IE*_*1*_	*ES*_11_	*IE*_*5*_	*ES*_52_
*IE*_*2*_	*ES*_21_	*IE*_*6*_	*ES*_61_
*IE*_*3*_	*ES*_31_	*IE*_*7*_	*ES*_72_
*IE*_*4*_	*ES*_41_		

### Design verification of medicinal bath water heater

**Figure 10:**
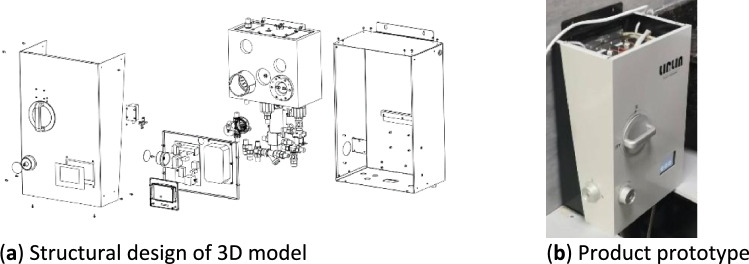
Design process for optimized product.

The design plan and prototype of the medicinal bath water heater created according to the technical elements of the optimal technical program are shown in Fig. 10.

**Figure 11:**
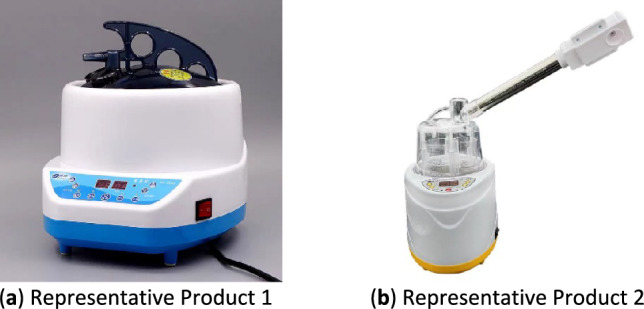
Representatives of existing products.

To evaluate the efficacy of the design scheme proposed in this study, we selected the top two bestselling medicinal bath equipment products from the Chinese e-commerce platform “Taobao” as representatives for comparison with our product. Figure 11 illustrates these two selected products.

Thirty respondents, randomly chosen from those who completed the Kano questionnaire in our study, participated in a medicated bath procedure using the product from our study as well as each of the two available products in the market. Following this, they completed the research questionnaire.

The process of a medicated bath involves these steps: Filling the tub with water, adding medicinal packets, Boiling the medicinal liquid, Performing the medicated bath, and cleaning the equipment.

**Figure 12:**
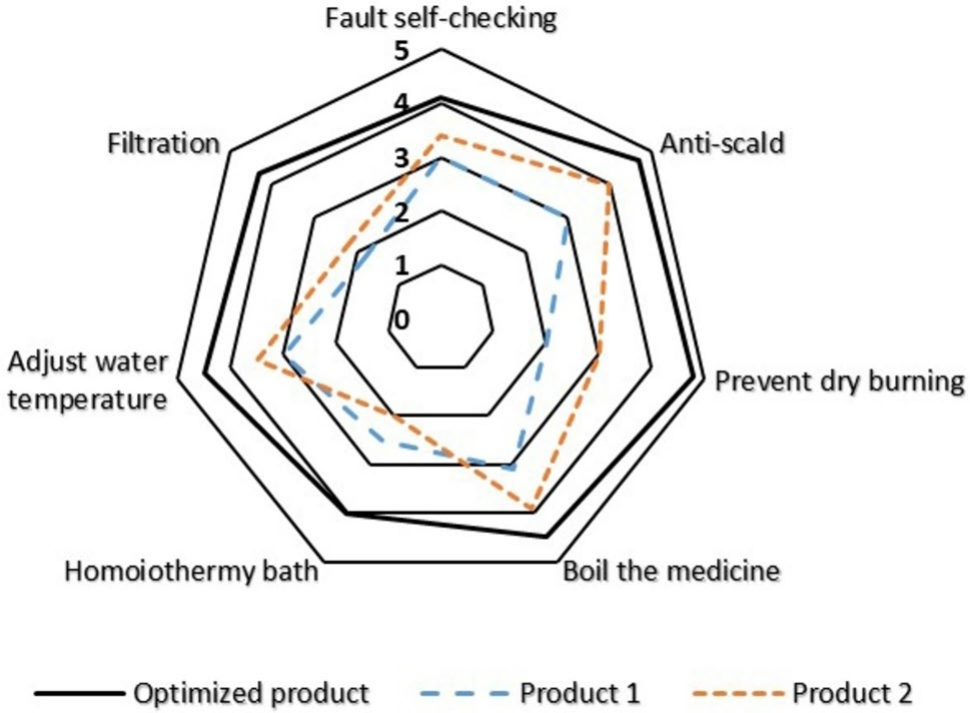
User satisfaction comparison.

The research questionnaire is designed based on the high-value technology elements (*IE*_*l*_) outlined in Table 9. Respondents are queried about their satisfaction with product functions aligned with each high-value technology element. Scores on the questionnaire, which utilizes a 5 point scale, positively correlate with satisfaction levels. The questionnaire results were compiled to calculate the average scores, depicted in Fig. 12. It's evident that user satisfaction levels with the medicated bath water heater have significantly improved compared to existing market products.

## Discussion and conclusions

### Discussion

The generation and decision-making of product technology solutions under complex user requirements have garnered significant research attention. Product development is a fusion of perceptual cognition and engineering technology, so the complexity of it also deserves in-depth study by scholars. This study concentrating on the conceptual design stage, excluding iterative updates and recycling in later stages. This study demonstrates that it is possible to split the product development process into different phases for study in order to gain a clearer understanding of the parts where there is room for optimisation, and to propose an integrated optimisation method based on this. The methodology is used to generate and decide on technical solutions for products that fulfil complex user needs, providing insights into translating user needs into product design solutions. This contribution helps to further promote user needs-driven product design research.

Compared with traditional design methods, the user requirement-driven product design method proposed in this study has certain advantages, such as accurately assessing design priorities by analysing user requirements, thus providing guidance for subsequent design and enabling the product to achieve higher user satisfaction.

This study analyses and calculations underscore that mitigating the impact of subjective factors in user needs analysis enhances accuracy. Additionally, incorporating multi-objective attributes into the product design decision can significantly enhance solution feasibility, aligning with prior research findings. Yi et al.^31^ employed a combined Kano-AHP approach to analyze user requirements, revealing that minimizing the impact of subjective factors in the analysis process enhances its accuracy. Chen et al.^32^ utilized multi-objective decision making in product styling design, yielding a solution with improved feasibility.

However, there are still some methodological shortcomings in this study, and there is still a certain degree of subjectivity in the translation of user requirements into technical programme using the KQGT model. Although this study improves the accuracy of the subjective analysis by means of expert group assessment, there are still some uncertainties, such as the transformation of high-value technical elements into technical solutions and design decision-making processes. For example, it relies on the subjective judgment and engineering experience of the expert group during the transformation of high-value technical elements into technical solutions. This may result in issues such as outdated technical solutions or reduced compatibility among them. And there are complex interrelationships behind these problems, which need to be considered comprehensively. As for the design decision, the uncertainty mainly comes from the establishment of evaluation objective. In this study, the principle of parallel design is used as the reference for the establishment of evaluation objective, but in the face of different market environments, the guidelines for the establishment of evaluation objective are also changing, so it is necessary to combine the product with the specific needs of the users and the market environment in the place where the product will be launched to set the evaluation objective.

In the subsequent research, we will construct a database of technical solutions for specific product technical problems, and further enrich the space and reliability of technical solution transformation by studying existing products and cutting edge technologies. At the same time, we will also study more efficient and accurate user requirements analysis methods to further improve the professionalism of the transformation of user requirements into product technical solutions, as well as to provide reference for the formulation of evaluation objective in the design decision-making, so as to improve the convenience and reliability of the composite product design model.

### Conclusions

With the aim of providing a technical solution for a product based on user demands, this paper proposed a product optimization design model (the KQGT model) driven by user requirements, which was verified by the investigation and development of a medicinal bath water heater as an example. The results showed that the optimized product technical solution effectively improved user satisfaction, and the main conclusions were as follows:

1. Combining the Kano model, dual analysis, and QFD for the qualitative and quantitative analysis of user requirements and technical elements can effectively facilitate the rational screening of user requirements and high-value technical elements, clarify the design focus, and improve user satisfaction with the technical solution.
2. We used game theory to optimize the technical programs, established the evaluation objectives of these programs through the principle of parallel design, and applied the overall utility value of the evaluation objectives as the decision-making index for solution optimization. This provided a theoretical basis for decision making in relation to multi-objective technical programs.

Despite the above advantages, this study did not investigate the transformation of user requirements into product appearance or interactive processes. In the future, we could attempt to comprehensively determine a product technical program and appearance scheme according to user requirements, so as to further improve the product optimization design model and form a systematic product design and decision-making method.

### Ethics statement

Ethical review and approval was not required for the study on human participants in accordance with the local legislation and institutional requirements.

### Statement of approval for human experiments

Identifies the institutional and/or licensing committee that approved the experiments, including any relevant details. Confirms that all experiments were performed in accordance with relevant named guidelines and regulations. Confirms that informed consent was obtained from all participants. All of the experimental procedures involving human were con-ducted in accordance with the Institutional guidelines of Shaanxi University of Science and Technology, China.

### Informed consent

Informed consent was obtained from all subjects and our legal guardian(s) involved in the research.

## Data Availability

The datasets generated and/or analysed during the current study are not publicly available due to privacy or ethical restrictions but are available from the corresponding author on reasonable request.
